# Twenty-One Flavors of Type 1 Innate Lymphoid Cells with PD-1 (Programmed Cell Death-1 Receptor) Sprinkles

**DOI:** 10.1093/discim/kyad003

**Published:** 2023-02-07

**Authors:** Katie J Smith, Giuseppe Sciumè, Shoba Amarnath

**Affiliations:** The Biosciences Institute, Newcastle University Biosciences Institute, Newcastle upon Tyne, UK; The Centre for Cancer, Newcastle University Centre for Cancer, Newcastle upon Tyne, UK; Medical School, Newcastle University, Newcastle upon Tyne, UK; Department of Molecular Medicine, Laboratory affiliated to Istituto Pasteur Italia – Fondazione Cenci Bolognetti, Sapienza University of Rome, Rome, Italy; The Biosciences Institute, Newcastle University Biosciences Institute, Newcastle upon Tyne, UK; The Centre for Cancer, Newcastle University Centre for Cancer, Newcastle upon Tyne, UK; Medical School, Newcastle University, Newcastle upon Tyne, UK

**Keywords:** ILC1, T-bet, EOMES, PD-1, LAG3, TIGIT

## Abstract

Innate lymphoid cells (ILCs) are tissue-resident immune cells that have been recently implicated in initiating and driving anti-tumor responses. ILCs are classified into three main groups, namely type 1 ILCs (ILC1), type 2 ILCs, and type 3 ILCs. All three groups have been implicated in either eliciting pro or anti-tumor immune responses in different cancer subtypes with the consensus that ILCs cannot be overlooked within the field of anti-tumor immune responses. In this review, we will specifically expand on the knowledge on ILC1, their characterization, function, and plasticity in anti-cancer immune responses. Within this premise, we will discuss caveats of ILC1 characterization, and expand on the expression and function of immune checkpoint receptors within ILC1 subsets, specifically focusing on the role of programmed cell death-1 receptor in controlling specific ILC1 responses. We summarize that ILC1s are a vital component in initiating anti-tumor responses and can be boosted by checkpoint receptors.

## Introduction

Innate lymphoid cells (ILCs) are tissue-resident innate immune cells that are parallel lineage to conventional T cells. The whole family of innate lymphocytes comprises of five groups: NK cells, helper ILC1s, type 2 ILCs, defined as natural and inflammatory ILC2s, type 3 ILCs including NCR^−^ ILC3s and NCR^+^ ILC3s and, finally, lymphoid-tissue inducer cells [1, 2]. NK cells are similar to CD8^+^ cytotoxic T cells in their ability to kill target cells while ILC1s, ILC2s, and ILC3s mirror CD4^+^ Th1, Th2, and Th17 cells, respectively [2]. ILC1s are defined by the master transcription factor T-bet and are activated by the presence of IL-12, IL-15, and IL-18 to mount an anti-viral and anti-tumor response by producing effector cytokines IFNγ and TNF-α [3, 4]. ILC2s are defined by GATA3 and are stimulated by IL-25, IL-33, and TSLP to secrete the cytokines IL-5, IL-9, and IL-13 to initiate parasitic defense and allergic responses [4]. ILC3s are defined by RORγt and the expression or not of natural cytotoxicity receptors (NCRs), and their functions are triggered in response to IL-23 and IL-1β leading to the production of IL-17, IL-22, and GM-CSF [4, 5]. ILC-derived cytokines can orchestrate the wider immune microenvironment such as driving T cell and myeloid/dendritic cell responses. In addition, they can use major histocompatibility complex (MHC) class II molecules for interaction with CD4^+^ T cells [6].

Despite not expressing antigen receptors, ILCs express co-receptors including a plethora of inhibitory and stimulatory receptors. These co-receptors include programmed cell death-1 (PD-1) and the ligand (PD-L1), inducible T cell co-stimulator (ICOS) and the ligand (ICOSL), and many others [6, 7]. These are key regulators of the immune system to prevent unwanted immune activation and are critical for maintaining peripheral tolerance [6]. However, there is currently a lack of understanding regarding the conditions under which these receptors are expressed on different ILCs in a variety of inflammatory environments.

ILCs are a heterogeneous population of innate immune cells due to their inherent ability to respond to alarmins, this heterogeneity further expands within each ILC subset [8]. In the peripheral blood of healthy individuals, between ~3000 and ~100 000, diverse phenotypes of NK cells exist [9]. Moreover, advances in single-cell RNA-sequencing technology have expanded our knowledge of NK cells’ transcriptional states in health and disease. Several NK cell states characterized by specialized gene expression profiles have been revealed in human melanoma metastasis [10]. Similarly, in human head and neck cancer, NK cells can differentiate into multiple cell states within the tumor, either highly active NK cells resembling intraepithelial ILC1s, or a hyporesponsive phenotype [11]. This data and many others published recently highlight the broad heterogeneity of innate lymphocytes in health and in cancer settings [12]. The innate phenotype of ILCs and the lack of antigen specificity means they can respond to a vast array of molecules therefore, perhaps the heterogeneity of ILCs is not so surprising.

The heterogeneity and plasticity of ILCs have resulted in the use of a plethora of transcription factors, cell surface markers, co-receptors, and alarmins to identify ILC populations. Although a standardization of ILC nomenclature was established by Spits *et al*., the current description of ILC subsets is complex and does not fit with all the novel ILC populations identified. Here we expand on this heterogeneity within ILC1 subsets and review the literature within this area of research.

## Defining ILC1

Typically, ILCs are grouped into subsets based on their phenotype, transcriptional requirement, and function. However, the expanding knowledge of the shared expression of markers and transcription factors present on ILCs and their substantial heterogeneity means this system is becoming much more complex and perhaps requires a more flexible classification. An open question in the field remains how to properly discriminate ILC1 from NK cells. Indeed, the ability of NK cells to acquire ILC1-like features, the identification of cytotoxic ILC1, and the novel array of ILC1 subsets expressing EOMES in humans and mice have challenged the current system of ILC classification [13, 14].

## NK cells vs. ILC1

NK cells are commonly defined as Lineage(lin)^−^CD56^+^cells in humans and CD3^−^NK1.1^+^NKp46^+^CD49b^+^ in mice [15]. Of note, NK1.1 is not expressed on NK cells of BALB/c mice but is expressed on C57BL/6 and SJL mice, with DX5 (CD49b) being the key marker for defining NK cells in BALB/c mice [16–18], highlighting strain-specific differences in NK marker expression. Historically, circulating human NK cells have been separated into three populations based on their differential expression levels of CD56 and CD16: CD16^hi^CD56^hi^, CD16^hi^CD56^dim^, and CD16^lo^CD56^hi^ [19]. At steady state, CD16^hi^CD56^dim^ represents the largest NK subset in peripheral blood [20], which is also considered a highly cytotoxic population [15]. However, more recently CD56 has been shown to be expressed on NKp44^+^ ILC3s [21], ILC2, ILC precursors which can give rise to CD117^−^ ILC3s [22], as well as on intraepithelial ILC1s [23]. As a result, CD56 represents a nonspecific substandard lineage marker for NK cells.

NK cells recognize MHC Class I molecules via the inhibitory receptors killer immunoglobulin-like receptors (KIR) in humans and lectin-like receptors of the Ly49 family in mice [24]. A plethora of activatory and inhibitory co-receptors are expressed within NK cell subsets. This is summarized in Table 1, in addition to a comparison of the ILC1 expression of these receptors [7, 25, 26].

**Table 1:** The expression of activatory and inhibitory receptors on murine NK cells and ILC1s

		NK cells	ILC1s
Activatory	CD28	+	+
	4-1BB	+	nd
	ICOS	+	nd
	NKp30	+	nd
	NKp44	+	+
	NKP46	+	+
	NKG2D	+	+
	DNAM-1	+	+
	KIR2DS1	+	nd
	KIR2DS2	+	±
	KIR2DS4	+	nd
	KIR2DS5	+	nd
	KIR3DS1	+	nd
	KIR2DL4	+	nd
Inhibitory	KIR2DL1	+	nd
	KIR2DL2/3	+	±
	KIR2DL2	+	nd
	KIR2DL5	+	nd
	NKG2A	+	±
	PD-1	+	±
	CTLA-4	+	±
	TIGIT	+	±
	CD94	+	nd
	LAG-3	+	±
	TIM-3	+	±

Note: + denotes expressed, − denotes not expressed, ± denotes under some conditions, and nd denotes not determined (not investigated). Refs. [5, 6, 15, 21, 28, 29, 31, 38, 40–44, 46, 47, 51, 66, 71, 93, 94].

In recent years, significant work has been performed on understanding what constitutes an NK cell vs. an ILC1. Expression of EOMES has been proposed as a marker to distinguish these cells in mice [27]. Indeed, NKp46^+^NK1.1^+^ cells in various murine tissues showed that typical NK markers were expressed on EOMES^+^ cells and ILC1 markers were expressed on EOMES^−^ cells [28]. However, it has been demonstrated in both humans and mice that intestinal intraepithelial ILC1 expresses EOMES [15, 29]. Similarly, a recent scRNA-seq study assessing ILCs from several tissues has revealed the presence of both ILC1 and ILC1-like cells characterized either by the selective expression of transcripts for EOMES or T-bet [30]. These studies emphasize the differences in group 1 ILCs within various tissues. A recent study by Lopes *et al.* [28] revealed extensive heterogeneity of NKp46^+^NK1.1^+^ cells between murine liver, spleen, salivary gland, and small intestine using CITE-seq analysis. NK cells in the spleen and liver were closely related to NK cells in the blood, while NK cells in the salivary glands and small intestine are more unique, for example, salivary gland NK cells express genes related to a mature, activated phenotype. The authors also revealed gene signatures that could be used to distinguish NK cells and ILC1s. Although both cell types expressed DNAM-1 and PD-1H (PD-1 homolog, also known as VISTA), ILC1s showed a much higher expression of these markers across all organs. Furthermore, syndecan-4 (SDC4) was expressed by almost all ILC1s but not by NK cells that makes this an intriguing marker to define these cell types [28]. Further investigation of protein expression in addition to RNA and functional analysis of all these populations are still required.

In line with the complexity described above concerning EOMES and T-bet expression in ILCs, Nagasawa and colleagues identified three subsets of ILC precursors in humans which have been defined as NKp46^+^, KLRG1^+^, and NKp46^+^ CD56^+^ cells [31]. When cultured for 5 days with IL-2 and IL-7, NKp46^+^ ILCs gave rise to ~20% of EOMES^+^ cells, expressing low levels of T-bet. However, the addition of IL-1β and IL-12 to NKp46^+^ ILCs highly upregulated T-bet and EOMES, and a high percentage co-expressed these two markers [31]. Thus, while IL-12 is a differentiation factor for human ILC1 in *in vitro* systems, this cytokine can drive NKp46^+^ ILC precursors to differentiate into both EOMES and T-bet expressing ILC1s.

The distinction between ILC1 and NK cells by the expression of IL7R has also remained elusive. In humans and mice, IL7R is predominantly expressed by ILC1s but not NK cells [30] and T-bet^+^ IFNγ^+^ ILCs express high levels of IL7R in humans in tonsil and inflamed intestine [21]. The mouse and the human liver instead appear quite heterogeneous for the expression of this receptor, with hepatic CD3^−^CD49a^+^CD56^+^ cells, resembling murine ILC1, which do not express IL7R [32]. Therefore, this suggests that IL7R needs to be further studied prior to being used as an appropriate marker for distinguishing ILC1s.

Although both NK cells and ILC1s are considered part of the innate immune system, both have been shown to display memory-like features. During cytomegalovirus infection, NK cells acquire a memory phenotype exhibiting a recall response resulting in expansion upon re-encounter with antigen and secretion of high levels of IFNγ [33]. Similarly, adoptively transferred activated NK cells respond more strongly upon reactivation indicative of a memory phenotype [34]. Intriguingly, IL-12/15/19-preactivated NK cells display a similar memory phenotype and are able to demonstrate anti-tumor effects in irradiated mice with melanoma [35, 36]. More recently, it has been discovered that ILCs possess long-term memory [37]. Following hapten sensitization, IL-7Rα^+^ ILC1s originate in the lymph nodes where they acquire memory and are recruited to the liver where they reside long-term. Long-lived memory ILC1s are characterized by the expression of CXCR6, IL7Rα, and CXCR3 [38]. Similarly, during allergic lung inflammation, ILC2s possess a memory-like signature similar to memory T cells [39]. These memory-like features emphasize the heterogeneity of group 1 ILCs, and future work will likely aid the understanding of the functional differences between NK cells and ILC1s.

Within this review, we will focus on the various subsets of helper ILC1s defined in normal and cancer immune microenvironments and expand on the importance of immune checkpoint receptor expression in ILC1s.

## ILC1s

**Figure 1:**
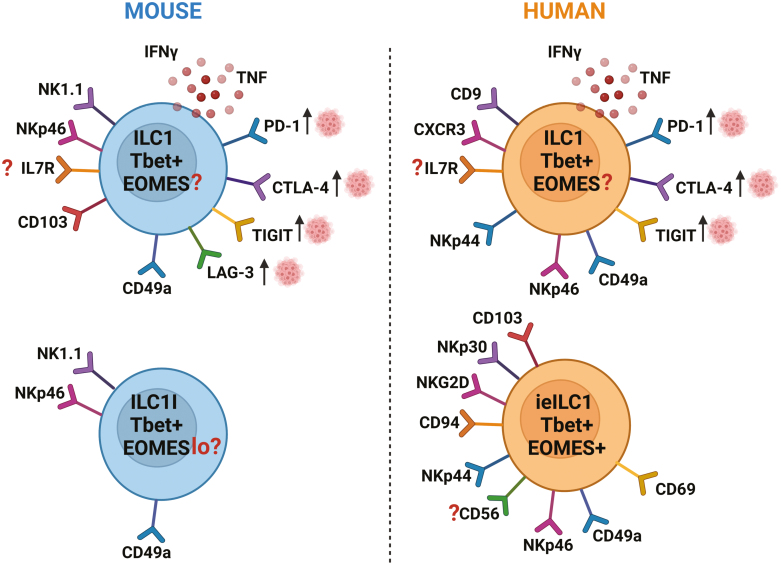
The variation in cell marker and transcription factor expression in ILC1s in mice (left panel) and humans (right panel). ? denotes conflicting data on marker expression in ILC1s. A variety of co-receptors are upregulated in the TME. Abbreviations: ILC1, innate-lymphoid cell 1; ILC1l, type 1 innate-like ILC1; ieILC1, intraepithelial ILC1. Refs [5, 6, 15, 21, 29, 31, 38, 40–43, 46, 51, 71].

Murine ILC1s are characterized as Lin^−^NK1.1^+^NKp46^+^CD49a^+^T-bet^+^RORγt^−^Eomes^−^ [6, 40, 41]. Defining human ILC1s is much more complicated, however, studies have shown these cells are Lin^−^CRTh2^−^CD117^−^IL7R^+^T-bet^+^RORγt^−^Eomes^−^CD9^+^CXCR3^+^ [5, 21, 31, 38, 42, 43] (Figure 1 and Table 2). ILC1s can also be defined by their production of IFN-γ and TNF-α in response to IL-12 and IL-15 stimulation [21, 29, 44] and human IFNγ-producing ILC1s express high levels of T-bet [45]. Moreover, there is a population of ILCs within the epithelial layer of the mucosa named intraepithelial ILCs that share many phenotypical traits with NK cells. In humans, these are defined by the specific expression of integrin chains (CD49a, CD103), NCRs (NKp30, NKp44, NKp46), and expression of both T-bet and EOMES [15, 29, 43, 46] (Figure 1 and Table 2). Distinct populations of intraepithelial ILCs express inhibitory KIRs (2DL and 3DL), although it is undetermined whether these populations express activating KIRs [15]. Most ILC1 characterization studies are yet to be fully translated to the tumor microenvironment and in this review, we have expanded on known ILC1 characterization, plasticity, and function within the tumor microenvironment below.

**Table 2:** : The expression of cell markers, co-receptors and transcription factors on mouse and human ILC1 subsets in health and cancers

	Mouse			Human		
	Steady-state ILC1	Type 1-like ILCs Dadi *et al.* 2016	ILC1 MCA1956 tumor Gao *et al.* 2017	Steady-state ILC1	Intraepithelial ILCs	ILCs CLL de Weerdt I *et al.* 2016
Lin	−	−	−	−	−	−
T-bet	**+**	**+**	**+**	**+**	**+**	nd
EOMES	−	lo	−	−	**+**	nd
RORγt	−	nd	nd	−	−	nd
IL7R	**+**	−	**+**	**+**	−	**+**
NK.1.1	**+**	**+**	**+**			
CD56	−	nd	nd	−	**+**	nd
CRTh2	−	nd	nd	−	−	−
CD103	±	nd	nd	−	**+**	−
CD117	−	nd	nd	−	−	nd
CD49a	±	**+**	**+**	**+**	**+**	nd
CD9	nd	nd	nd	**+**	nd	nd
CD69	±	nd	**+**	−	**+**	**+**
CD94	nd	nd	nd	−	**+**	−
NKp30	nd	nd	nd	**+**	**+**	nd
NKp44	**+**	nd	nd	**+**	**+**	nd
NKP46	**+**	**+**	**+**	**+**	**+**	nd
CXCR3	nd	nd	nd	**+**	nd	nd
PD-1	lo	nd	**+**	lo	nd	nd
CTLA-4	nd	nd	**+**	lo	nd	nd
TIGIT	nd	nd	**+**	**+**	nd	nd
LAG-3	nd	nd	**+**	**+**	nd	nd
TIM-3	nd	nd	−	**+**	nd	nd

Note: + denotes expressed, − denotes not expressed, ± denotes under some conditions, nd denotes not determined (not investigated). Refs. [5, 6, 15, 21, 29, 31, 38, 40–43, 46, 51, 66, 71].

## ILC1 in cancer

ILC1s are tissue-resident innate lymphocytes and thus may have a prominent role in detecting malignant cells and directing anti-tumor responses by reacting to changes within the microenvironment and by orchestrating the recruitment of other immune cells [47]. In humans, T-bet^+^ ILC1s are enriched in patients with colorectal cancer; these cells were also identified in gastrointestinal and breast tumors, although T-bet was not measured [48–50]. However, their immunological role in these cancers remains unclear.

Tumor immunosurveillance by ILCs has been proposed for type-1-like ILC1s which are defined as TCR^-^NK1.1^+^CD49a^hi^T-bet^+^EOMES^lo^IL7R^-^NKp46^+^ (Figure 1 and Table 2). These cells were described as ILC1-like cells because they lacked IL7R and expressed EOMES, which is usually absent on ILC1 [51]. Once again, this emphasizes the inconsistencies in an expression of proposed conventional ILC1 markers within the TME.

Activating KIR receptors bind ligands that are induced during cellular stress and tumor transformation, and inhibitory KIR receptors bind MHC class I molecules expressed by healthy cells; therefore, it is likely that ILC1s expressing these receptors are able to detect and respond to cancerous cells [47]. Moreover, ILC1 constitutively expresses the activating receptor NKG2D, thus it is possible that ILC1 are interacting with NKG2D ligands expressed by stressed or malignant cells [47, 51]. Mice lacking NKG2D have increased expression of NKG2D ligands and increased development of aggressive early tumors in a prostate cancer model [52]. Whether and how ILC1 contribute to restrain cancer growth via activating receptor is still poorly investigated, due to the lack of mouse models selectively targeting these cells, without affecting NK cell functions. The rapid response of ILCs to pre-cancerous cells, and their expression of a wide range of activating and inhibitory receptors provides evidence for ILCs as first responders to tumor alarmins.

A wide array of activating receptors described in Table 1, including NKp46 (encoded by the *Ncr1* gene) is expressed on ILC1s and NK cells. The anti-tumor action of NK cells is dependent on these receptors. Mice lacking this receptor showed increased metastasis and worse survival [53]. Of note, conditional knock-out mice based on *Ncr1-cre* deletion not only affects NK cells but also ILCs (ILC1 and NCR^+^ ILC3), therefore it is likely that ILCs contribute to anti-tumor responses, although this requires more specific depletion of ILC1s to establish their direct role [47]. The extensive heterogeneity of NK cells in cancer has recently been shown by single-cell RNA-sequencing analysis of human melanoma tumors which revealed 7 NK clusters with specific gene expression profiles [10]. Of note, the markers used for the isolation of NK cells in this study are also expressed on ILCs. This suggests dynamic functional specialization of NK cells in cancer, and most likely ILC1s, and also highlights the diverse range of phenotypes group 1 ILCs can adopt. Further understanding of the expression of these receptors and how the receptors are dysregulated in the TME could lead to harnessing NK cells and ILC1s for cancer treatment. This is extensively reviewed in Ref. [25].

Our understanding of regulatory mechanisms in inflammatory conditions other than cancer may indicate mechanisms by which ILC1s are involved in cancer. During early cytomegalovirus infection, IFNγ is predominantly produced by ILC1s within the infected tissue and this precedes the activation of NK cells [54]. Thus, this shows ILC1 activation is rapid and supports the notion that ILC1s respond early to tumor alarmins, before NK cells and conventional T and B cells.

### Anti-tumor action

In mice, tissue-resident tumor-associated ILC1 expand in pre-cancerous lesions of PyMT mice and showed cytotoxicity toward tumor cells [51]. IL-15 has been suggested to be a stress-induced alarmin that signals to ILCs to kill cancerous cells. Indeed, the loss of IL-15 reduces the cytotoxic ILC pool and accelerates tumor growth, although the various source of IL-15 is currently under investigation [47, 51]. It is possible that stromal cells could be a source of IL-15 as they have been shown to express IL-15 in secondary lymphoid organs [55]. There is also evidence that tumor cells could be a source of IL-15. In colorectal cancer, IL-15 was produced by tumor cells shown by immunohistochemical analysis [56], and in a murine breast cancer model, IL-15 was produced by cancer cells and functioned as an alarmin [57]. Moreover, it has been shown that ILC1, together with NK cells, contributes to limit tumor growth in distinct mouse models of liver metastasis [58]. In these settings, tissue-resident ILC1 was suggested to control metastatic seeding, while NK cells restrained tumor growth.

ILC1s express several molecules driving cytotoxicity including granzyme A, granzyme B, granzyme C, and TRAIL, and are highly efficient in killing tumor cells [51, 59–61] demonstrating a potent immunosurveillance and killing mechanism of ILCs. Moreover, ILC1 upregulates a selection of chemokine receptors and cytokines. They secrete IFNγ in response to IL-12 within the TME which can exert anti-tumor affects by inducing tumor cell death and preventing tumor growth [62, 63]. ILC1 also upregulates CXCR6, CCR5, CXCR3, and CCR1 which can recruit RORγt^+^ ILC3s to the tumor leading to the inhibition of tumor growth [64]. Similarly, in colorectal cancer intraepithelial T-bet^+^EOMES^+^ ILC1s produced IFN-γ and expressed granzyme B and perforin which can inhibit tumor growth [64, 65]. Therefore, ILC1s can be involved in direct killing, immunosurveillance, and recruitment of other immune cells to promote anti-tumor responses.

### Pro-tumor action

In addition to anti-tumor responses, ILC1 has been shown to promote tumor growth. In chronic lymphocytic leukemia (CLL), ILC1 was elevated in the peripheral blood compared to healthy controls [66]. This study showed, *in vitro*, that ILCs cultured with CLL cells show differential expression of TNF-α, however, a direct role for ILC1s was not revealed. Moreover, in this study, total ILCs were defined as Lin^−^ IL7R^+^CD161^+^ and ILC1 was characterized as CRTH2^-^CD117^−^. Of note, in this population, T-bet has not been measured thereby it is possible that this could be a precursor or an improperly differentiated NK cell derivative.

Stimulated ILC1s from CLL patients had significantly reduced production of TNF, although similar levels of CD69 expression and IFN-γ production were noted. The authors stimulated ILC1s from patients with PMA/ionomycin and cultured them with CLL cells and ILC1s were unable to enhance IFN-γ or TNF production showing a defect in responsiveness of ILCs from cancer patients which suggests an immunosuppressive role for ILC1s in certain cancer environments [66].

In acute myeloid leukemia (AML), there is a significant enrichment of ILC1s in peripheral blood compared to healthy controls and a significant reduction in the production of IFN-γ, TNF, and IL-5/IL-13 in total ILCs defined as Lin^-^IL7R^+^ [67]. The authors did not isolate ILC1s in patients to investigate subset cytokine production; however, it is likely that ILC1s within this pool show decreased IFN-γ and TNF secretion. As these cytokines have anti-tumor affects it is possible that a reduction in their production in AML patients results in pro-tumor affects, but this is currently unknown and requires further investigation.

Finally, ILC1s are increased in number and are pathogenic in inflammatory bowel disease (IBD). Patients with IBD have an increased risk of developing colorectal carcinoma which has led to the suggestion that ILC1s may be involved in colorectal carcinoma development [47]. However, the direct role of ILC1s during IBD-associated colorectal carcinoma is undetermined. It is unknown whether ILC1s have an anti- or pro-tumor role in this disease, and it is possible that there is a broad ineffective immune response contributing to the increased risk of cancer.

In bacteria-induced colon cancer, IL-17 and IL-22-producing colonic ILCs are involved in driving pro-tumor responses; specifically, IL-22 sustains inflammation during cancer by STAT3 phosphorylation in epithelial cells inducing proliferation [68]. Recently, in humans, ILC2s have been shown to be associated with improved tumor burden in CRC [69]. There could be a potential mechanism operational here whereby the presence of alarmins namely IL-1β may enhance ILC2-ILC1 conversion [70], thereby participating in CRC-driven ILC pro-tumor function. Indeed this process within CRC is yet to be reported. Therefore, it is possible that there are pro-tumor actions of other ILC subsets in colon cancers.

### Angiogenesis

Similar to the pro-tumor functions of ILC1s, there is also evidence suggesting that these cells can promote angiogenesis. ILC1s infiltrating mouse fibrosarcoma tumors show an upregulation of genes involved in angiogenesis [71] and express high levels of TNFα that increases vascular cell adhesion molecule expression and angiogenesis. However, TNF has also been shown to damage tumor vasculature [64]. Similarly, ILC1s secrete IFN-γ in the TME which is anti-angiogenic [64]. Therefore, the angiogenetic effects of ILC1-derived cytokines require further investigation.

### Lessons learnt from NK cells

Our understanding of how NK cells respond to the TME may provide insight into the additional role of ILC1s in cancer progression. NID1 ligand expression is increased in ovarian cancerous tissue and is recognized by the NKp44 receptor expressed on NK cells within the TME. This receptor–ligand interaction is thought to increase NK cell recognition of malignant cells [72] and has been shown to promote the migration, invasion, and resistance of ovarian cancer cells and metastasis [73]. Interestingly, soluble NID1 reduces cytokine production and cytotoxicity from NK cells, whilst membrane-bound NID1 increases the production of IFN-γ [74]. Therefore, this provides a rationale for targeting NID1-NKp44 interaction as a potential therapeutic. Similarly, prostaglandin E2 produced by thyroid cancer cells downregulated the expression of NKp44 and NKp30, leading to a reduction in NK cell cytotoxicity and maturation [75]. ILC1s express multiple activating NK receptors therefore, similar interactions may occur with ILC1 within the tumor to impact the cytotoxicity and anti-tumor function of ILCs. This also highlights the importance of co-receptor interactions during cancer and the potential for these receptors to be targeted for cancer treatment.

Thus, within the tumor, ILC1s rapidly respond to alarmins that are derived from infiltrating immune cells, epithelial cells and stromal cells that allow them to carry out anti- and pro-tumor functions. Additionally, there is now increasing evidence of the interplay between ILC1s and other immune cells within the TME. This will not be addressed in this review but has been reviewed extensively in Ref. [50].

## ILC1 plasticity within the TME

Despite ILCs being categorized into different subsets, their heterogeneity renders this population extremely plastic under perturbed immunity [76–78]. The heterogeneity and plastic abilities of ILCs explain the pleotropic roles of ILCs in cancer. It is important to understand the triggers as well as mechanisms underlying recognition that initiate ILCs and the functional relevance in disease in order to harness these cells for translation into the clinic. Within the TME, the array of cytokines available to ILCs will induce the conversion of ILCs and allow for adaptation to changing environmental stimuli. In human hepatocellular carcinoma, NK cells can transition to tumor ILC1, both of which express T-bet and high levels of EOMES [79]. This was also described in a subcutaneous MCA1956 tumor model in mice, where TGF-β signaling in the tumor microenvironment resulted in the plasticity of CD49a^−^CD49b^+^Eomes^+^ NK cells into a CD49a^+^CD49b^+^Eomes^+^ intermediate type 1 ILC1 population and then CD49a^+^CD49b^−^Eomes^int^ ILC1s [71]. The NK cell and intermediate ILC1-like population had low levels of T-bet, but this was highly upregulated in ILC1s. Thus, conversion to alternative group 1 ILCs can occur in response to the TME.

Importantly, a role for the co-receptor PD1 in the plasticity of T cells has been demonstrated by us [80, 81]. Therefore, it would be unsurprising for co-receptors to also be involved in the plasticity and function of ILCs.

## Co-receptor expression on ILC1s within the TME

ILCs express a wide range of co-receptors within the TME that are involved in controlling the immune response. As mentioned, ILC1s have been shown to express significant amounts of LAG3, while modest expression of PD-1, CTLA-4, TIGIT, KLRG1, and GITR has been reported [7]. Many of these receptors are upregulated within the TME and are expressed at higher levels on ILC1s than on tumor-infiltrating NK cells, suggesting a role for co-receptors in rendering ILC1 inhibitory function in tumor [71]. A comprehensive review of co-inhibitory receptors that are expressed on ILC1s in TME is discussed below and further, we have highlighted studies where functional role of these co-inhibitory receptors has been investigated. While most studies report expression of co-inhibitory receptors, there is a significant paucity of data that defines the functional consequence of co-inhibitory receptor expression and its impact on ILC function, cytokine production, proliferation, and so on. In addition, it is unclear which alarmins induce these coreceptors on ILC1s in TME and the precise signaling pathways and molecular mechanisms by which the co-inhibitory receptors affect ILC1 function.

### CTLA4

CTLA-4 acts as a negative regulator of T cell activation by binding CD80 and CD86 with a much greater affinity than CD28 [82]. CTLA-4 is expressed at low levels in peripheral blood, but this is significantly upregulated in ILC1s infiltrating human malignant breast tumors [83], and melanoma [84], while, it is expressed at much lower levels in hepatocellular carcinoma [79]. Moreover, CTLA-4 is expressed on both ILC1 and NK cells in mouse tumor models, although ILC1 has higher expression than NK cells within the tumor [71]. Specifically, within transplanted MCA1956 tumors, ~80% of ILC1s express CTLA-4 [71], thereby suggesting an inhibitory axis that is controlled by coreceptors in ILC1s. However, in this study, although expression was noted, functional analysis was not performed to detect if CTLA4 participated in the ILC1 function.

Treatment with a CTLA-4-specific monoclonal antibody has been used as a cancer therapy for metastatic melanoma. It has been shown that antibody treatment in a murine model of melanoma reduces the number of CTLA-4^+^ T regulatory cells within the TME in an FcγR-dependent manner [85]. Therefore, it is possible that CTLA-4 monoclonal antibody treatment may result in ILC1 depletion. If this is the case, it would be interesting to elucidate the functional implications of ILC1 depletion during metastatic melanoma.

### TIGIT

TIGIT belongs to the immunoglobulin superfamily receptors and is expressed by group 1 ILCs [7]. Around ~60% of ILC1s infiltrating MCA1956 tumors express TIGIT [71]. Moreover, ILC1-like cells from colorectal cancer patients that are absent in normal mucosa are enriched in genes encoding for inhibitory receptors including TIGIT and TIGIT^+^ ILC1-like cells were identified by flow cytometry within tumors but not normal tissues [8]. It has been shown that TIGIT ligands CD115 and CD112 are expressed within tumors [86]. CD115 is upregulated in melanoma tissues [87] and colorectal cancers where it is involved in colon cancer progression [88]. Therefore, it is possible that ILC1s are directly interacting with cancer cells via TIGIT. However, similar to CTLA4, functional analysis was not performed to detect if TIGIT participated in ILC function, for example, whether blocking TIGIT enhanced ILC1 cytokine expression or proliferation was not tested. As TIGIT is an inhibitory receptor, it is likely that TIGIT, and expression of other inhibitory co-receptors on ILC1s, could result in suppression of ILC1 proliferation and activation, however, this is currently unknown.

### LAG-3

LAG-3 is expressed on activated T cells, regulatory T cells, B cells, and DCs [7]. In healthy humans, circulating ILC1s, but not ILC2s or ILC3s express LAG-3 [7]. Within the tumor, around half of ILC1s infiltrating MCA1956 tumors express LAG-3 [71]. Similarly, ~30% of ILC1-like cells that transdifferentiated from NK cells in a TGF-β dependent manner express LAG-3, interestingly very few NK cells expressed LAG-3 within the TME [71].

### PD1

PD-1 is an inhibitory receptor that binds PDL-1 and PDL-2 [89]. In mice during steady state, ILC1s express low amounts of PD-1 [7]. This is marginally upregulated in MCA1956 tumor-infiltrating ILC1s with only 5% of ILC1s expressing PD-1 [71]. PD-1 is also expressed on ILC1s infiltrating benign tumors, malignant breast tumors, and malignant GI tumors, although expression is not altered compared to peripheral blood [83]. Studies have shown that in hepatocellular carcinoma PD-1 expression increases within tumor tissue compared to non-tumor tissues [79] and non-small cell lung cancer tumor tissue, up to ~50% of ILC1s express PD1 [90]. However, none of these studies have reported the alarmins that can induce PD-1 on ILC1s and whether PD-1 participates in dampening the ILC1 function within the TME. In this context, recent evidence shows that NK cells can acquire PD-1 directly from leukemia cells via trogocytosis, instead of being endogenously expressed, providing further complexity in the mechanisms underlying the inhibition of NK cell responses [91]. Whether trogocytosis can lead to the acquisition of PD-1 expression on ILC1 remains to be investigated.

Recently, our group has identified a previously undefined subset of T-bet^+^NK.1.1^−^ NKp46^–^ ILC1s that is regulated by PD1 in the TME in both murine and human cancers [92]. Tumor-derived lactate was shown to induce PD-1 within these subsets and PD1 signaling inhibited the proliferation of T-bet^+^ ILC1s in the TME along with reducing IFN-γ production thereby promoting pro-tumor responses. Therefore, PD1 could be a primary regulator of this new ILC subset in the TME, whereas other co-inhibitory receptors, such as LAG3, could be primary regulators of conventional ILC1s [71].

### TIM-3

TIM-3 is a type 1 glycoprotein expressed on many different immune cells, including NK cells in a range of cancers [7]. However, in mice transplanted with MCA1956 tumors, TIM3 was not detected on NK cells or ILC1s [71].

Therefore, co-receptors are expressed on ILC1s within the tumor microenvironment in multiple cancer models. Importantly, 5% of group 1 ILCs express PD-1 compared to 30–70% expressing LAG3 and CTLA4 [71]. This supports the notion that ILCs upregulate multiple different co-receptors within the tumor that likely boosts the anti-tumor responses carried out by ILC1s, however without understanding the molecular mechanisms by which these co-receptors regulate ILC function, their expression remains correlative of immunosuppressive tumor microenvironment.

## Conclusion

To date, a significant proportion of our understanding of the phenotype of ILC subsets has heavily relied on transcriptomic data. Although single-cell RNA sequencing generates high-resolution data about each cell type, it does not give information regarding the protein expression in the cell. Therefore, it is important to consider that transcription does not always result in translation, and the presence of cell markers and transcription factors at the protein level is required to effectively define ILC subsets.

Within the TME, we propose that ILCs are the first to respond to tumor alarmins. These alarmins drive the expression of co-inhibitory receptors such as PD-1, which are able to promote anti-tumor responses and encourage the plasticity of ILC subsets. We hypothesize that patients who cannot upregulate PD1 may upregulate other co-receptors, but this is not clearly understood. Future work should focus on which co-receptors compensate for PD1 deficiency in order to target multiple co-receptors for cancer treatments. However, for this to occur a clearer understanding of the expression of cell markers on different ILC subsets is required as currently this is extremely complex and requires a standardization of mouse and human ILC1 nomenclature. Reviewing the literature suggests that T-bet was expressed in all ILC1 populations and perhaps the best marker for defining ILC1 function and plasticity.

## Abbreviations

AML: acute myeloid leukemia
CLL: chronic lymphocytic leukemia
CTLA-4: cytotoxic T-Lymphocyte Associated Protein 4
ICOS: inducible T cell co-stimulator
ICOS-L: inducible T cell co-stimulator ligand
ieILC1: intraepithelial ILC1
IFNγ: interferon gamma
ILC: innate lymphoid cell
ILC1l: type 1 innate-like ILC1
LAG-3: Lymphocyte activation gene-3
NCR: natural cytotoxicity receptors
NK: natural killer
PD-1: programmed death receptor-1
PD-L1: programmed death receptor-ligand 1
TGF: Transforming growth factor
TIGIT: T cell immunoreceptor with Ig and ITIM domains
TME: tumour microenvironment
TNF: tumor necrosis factor
